# Hypoxia‐induced secretion stimulates breast cancer stem cell regulatory signalling pathways

**DOI:** 10.1002/1878-0261.12500

**Published:** 2019-06-26

**Authors:** Hanna Jacobsson, Hannah Harrison, Éamon Hughes, Emma Persson, Sara Rhost, Paul Fitzpatrick, Anna Gustafsson, Daniel Andersson, Pernilla Gregersson, Ylva Magnusson, Anders Ståhlberg, Göran Landberg

**Affiliations:** ^1^ Department of Pathology and Genetics Institute of Biomedicine Sahlgrenska Cancer Center University of Gothenburg Sweden; ^2^ Breakthrough Breast Cancer Unit Centre for Molecular Pathology Institute of Cancer Sciences Paterson Institute for Cancer Research University of Manchester UK; ^3^ Manchester Cancer Research Centre The University of Manchester UK; ^4^ Wallenberg Centre for Molecular and Translational Medicine University of Gothenburg Sweden; ^5^ Department of Clinical Pathology and Genetics Sahlgrenska University Hospital Gothenburg Sweden

**Keywords:** breast cancer, cancer stem cells, hypoxia, IL‐12, IL6, JAK‐STAT, secretion

## Abstract

It is well known that tumour cells are dependent on communication with the tumour microenvironment. Previously, it has been shown that hypoxia (HX) induces pronounced, diverse and direct effects on cancer stem cell (CSC) qualities in different breast cancer subtypes. Here, we describe the mechanism by which HX‐induced secretion influences the spreading of CSCs. Conditioned media (CM) from estrogen receptor (ER)‐α‐positive hypoxic breast cancer cell cultures increased the fraction of CSCs compared to normal growth conditions, as determined using sets of CSC assays and model systems. In contrast, media from ERα‐negative hypoxic cell cultures instead decreased this key subpopulation of cancer cells. Further, there was a striking overrepresentation of JAK‐STAT‐associated cytokines in both the ERα‐positive and ERα‐negative linked hypoxic responses as determined by a protein screen of the CM. JAK‐STAT inhibitors and knockdown experiments further supported the hypothesis that this pathway is critical for the CSC‐activating and CSC‐inactivating effects induced by hypoxic secretion. We also observed that the interleukin‐6‐JAK2‐STAT3 axis was specifically central for the ERα‐negative hypoxic behaviour. Our results underline the importance of considering breast cancer subtypes in treatments targeting JAK‐STAT or HX‐associated processes and indicate that HX is not only a confined tumour biological event, but also influences key tumour properties in widespread normoxic microenvironments.

**Abbreviations**
BCSC: breast cancer stem cell
CM: conditioned media
CSC: cancer stem cells
DMEM: Dulbecco modified Eagle′s medium
ERα: estrogen receptor‐alpha
HIF: hypoxia‐inducible factor
HX: hypoxia
IL: interleukin
NX: normoxia
qPCR: quantitative PCR
RPMI: Roswell Park Memorial Institute
scr: scramble

## 1 Introduction

Breast cancer represents a diverse group of malignancies derived from ductal epithelium. In recent years, the microenvironments of these cancers have been shown to play a critical role in tumour progression as well as therapeutic response (Hu *et al*., 2009; Tyan *et al*., 2011). Importantly, different subtypes of breast cancer demonstrate contrasting direct effects towards a hypoxic environment (Fuchs *et al*., 2004; Korkaya *et al*., 2011). Our group has previously shown that reduced oxygen levels have an opposing, direct estrogen receptor (ER)‐α‐dependent effect on breast cancer stem cell (BCSC) populations (Harrison *et al*., 2013). Given that many reports highlight secretion as a mean for tumour cells to communicate within the tumour microenvironment, we hypothesized that hypoxic regions secrete factors to nearby cells and thereby influence cellular plasticity and expansion or reduction of stem cell properties (Harrison *et al*., 2018; Hu *et al*., 2009; Tyan *et al*., 2011).

Furthermore, breast cancer consists of heterogeneous cell compositions explained by clonal variation within the tumour as well as an intrinsic balance between different cellular differentiation stages, including cancer stem cell (CSC) pools and more differentiated cells (Campbell and Polyak, 2007; Marusyk and Polyak, 2010). These subpopulations of tumour cells with specific qualities represent a major challenge for treatment resistance in general as well as for monitoring therapy efficacy. Regarding clonal variations, characterization through, for example, sequencing of distal parts of tumour has highlighted the complexity and has identified driver mutations leading to clonal expansion (Vogelstein *et al*., 2013). Nevertheless, within this study we focused on cellular differentiation aspects of heterogeneous cellular populations.

Previously, much research focus has been on trying to understand inflammatory responses within the tumours through tumour interactions with the extracellular matrix, mesenchymal stem cells, immune cells, cancer‐associated fibroblasts and tumour‐activated macrophages (Criscitiello *et al*., 2014; Gilkes *et al*., 2014; Ginestier *et al*., 2010, 2011; Li *et al*., 2012; Lu *et al*., 2014). A crosstalk between CSCs and their microenvironment via certain cytokines has been demonstrated (Bak *et al*., 2016; Di Stefano *et al*., 2010; Sansone *et al*., 2007; Singh *et al*., 2013). In 2011, Liu and coworkers reported that BCSCs are regulated by cytokine‐related signalling pathways and highlighted the influence IL6 and IL8 production to maintain CSC properties (Liu *et al*., 2011; Sansone *et al*., 2007). Additionally, IL6 has been shown to function as a molecular switch between non‐CSCs and CSCs in breast cancer, suggesting a dynamic system that is dependent on communication between the different cell types through secretion to maintain the CSC niche (Iliopoulos *et al*., 2011).

## 2 Material and methods

### 2.1 Cell lines

MCF7, T47D, MDA‐MB‐231, and MDA‐MB‐468 were purchased from American Type Culture Collection (Manassas, VA, USA). Cell lines were authenticated by multiplex PCR assay using the AmpF/STR system (Applied Biosystems, Thermo Fisher Scientific, Waltham, MA, USA) and confirmed as mycoplasma free. Monolayers were grown in Dulbecco modified Eagle′s medium (DMEM; Lonza, Basel, Switzerland; DMEM/10% FBS/2 mmol/l‐glutamine/1% penicillin/streptomycin for MCF7 and CAL120 cells) or Roswell Park Memorial Institute (RPMI) medium (Fisher Scientific, Waltham, MA, USA; RPMI/10% FBS/1% sodium pyruvate/2 mmol·L^−1^ l‐glutamine/1% penicillin/streptomycin for T47D, MDA‐MB‐231 and MDA‐MB‐468 cells). Cells were maintained in a humidified incubator at 37 °C at an atmospheric pressure of 5% (v/v) CO_2_/air.

### 2.2 Hypoxic cell culture

Cells were incubated for 48 h in the SCI‐tiveN hypoxic workstation (Ruskinn, Sanford, ME, USA) in 1% O_2_, 5% CO_2_ and 94% N_2_ in a humidified environment at 37 °C. Cells were seeded, cultured and harvested within the workstation to maintain hypoxia (HX) at all times. Confirmation of hypoxic conditions was carried out using western blot analysis of HX‐inducible factor (HIF)‐1α protein expression.

### 2.3 Mammosphere culture

Mammosphere culture was carried out as previously described (Shaw *et al*., 2012). In brief, following treatment, cells were resuspended as single cells using a 25‐gauge needle. A total of 5000 cells per 2 mL media were seeded into Polyhema‐coated 6‐well culture plates in MEM (Gibco™, Thermo Fischer Scientific; phenol red free) containing 1% penicillin/streptomycin, 200 μg human recombinant EGF (VWr) and 1% B27 supplement (Life Technologies, Thermo Fischer Scientific). Cells were maintained for 5 days in a humidified incubator at 37 °C at an atmospheric pressure of 5% (v/v) CO_2_/air and analysed for mammosphere formation by microscope. Mammospheres ≥ 50 μm were counted.

### 2.4 Holoclone culture

Holoclone culture was carried out as previously described (Liu *et al*., 2013). In brief, following treatment, cells were resuspended as single cells using a 25‐gauge needle. A total of 500 cells per 2 mL media were seeded into six‐well culture plates in DMEM (Lonza; DMEM/10% FBS/2 mmol·L^−1^ l‐glutamine/1%penicillin/streptomycin for MCF7 cells) or RPMI medium (Fisher Scientific; RPMI/10% FBS/1% sodium pyruvate/2 mmol·L^−1^ l‐glutamine/1% penicillin/streptomycin for T47D and MDA‐MB‐231 cells). Cells were maintained for 5–10 days in a humidified incubator at 37 °C at an atmospheric pressure of 5% (v/v) CO_2_/air and analysed for colony formation by microscopy.

### 2.5 Patient *ex vivo* explants

Breast cancer tissue was obtained after written informed consent through Sahlgrenska University Hospital, Gothenburg, Sweden. 500 μm thick sections from breast cancer tumours were prepared at Department of Pathology at Sahlgrenska University Hospital using a vibrating blade microtome LEICA VT1000S and cultured in duplicate on a presoaked gelatin sponge (Johnson and Johnson, New Research, New Brunswick, NJ, USA) in six‐well plates containing DMEM‐F12 (Lonza; 10% FBS/1% PenStrep) (Centenera *et al*., 2013; Dean *et al*., 2012) without covering the tissue. Tissues were cultured in either normoxic (21% O_2_) or hypoxic (1% O_2_) conditions at 37 °C for 48 h and then formalin‐fixed and paraffin‐embedded or preserved in RNAlater (Invitrogen, San Diego, CA, USA). Conditioned culture media were taken from explants and centrifuged at 300 rcf for 5 min to remove cellular debris. The conditioned media (CM) were then used to either treat cell lines or analysed using western blot.

### 2.6 Human antibody array

Culture media from 48 h normoxic (21% O_2_) or hypoxic (1% O_2_) conditions at 37 °C were collected from ERα‐positive (MCF7) and ERα‐negative (MDA‐MB 231) cell cultures and analysed for protein secretion using human antibody array (RayBiotech, Inc., Norcross, GA, USA). The antibody array was performed according to the protocol provided by the manufacturer.

### 2.7 Western blotting

Cells were lysed in RIPA lysis buffer containing 50 mm HEPES, 150 mm NaCl, 1 mm EDTA, 1% (w/v) CHAPS, and protease and phosphatase inhibitor cocktail (Sigma‐Aldrich, St. Louis, MO, USA). Subsequently, the cell lysates were boiled in 2× SDS/PAGE loading buffer for 10 min, and then, 25 μg of protein was separated on a 4–20% SDS/PAGE and transferred to Hybond‐C Extra nitrocellulose membrane. Primary antibodies used in the experiments were β‐actin (ab8226; Abcam, Eugene, OR, USA), ERα (M7047; DAKO), HIF‐1α (#61095,9; BD Biosciences, San Jose, CA, USA), Phospho‐Stat3 (Tyr705; 9131; Cell Signalling), STAT3 (D3Z2G; Cell Signalling, Danvers, MA, USA), and PR (M3569; DAKO, Agilent, Santa Clara, CA, USA). HRP‐conjugated secondary antibodies were purchased from Life Technologies. Protein detection was carried out using ECL system (GE Healthcare, Chicago, IL, USA).

### 2.8 Transient transfections, inhibitor treatment, and cytokine treatment

MCF7 and T47D cells were transfected with 25 nm small interference RNA (siRNA) against the ESR1 gene (Origene, Herford, Germany) using the transfection reagent Viromer blue (Lipocalyx, Halle, Germany). In brief, cells were seeded in a six‐well plate 1 day prior to transfection and incubated at 37 °C and 5% CO_2_ overnight. The transfection was carried out in normal complete growth media. Cells were then incubated for 48 h in either normoxic (21% O_2_) or hypoxic (1% O_2_) conditions. CM media were collected and spun down at 300 rcf for 3 min to remove cell debris and dead cells. Overexpression of ERα with VP16ERα vector (Addgene plasmid^27^, Watertown, MA, USA) in MDA‐MB 231 cells was carried out using Lipofectamine 2000 (Invitrogen) according to manufacturer′s description. VP16 empty vector was used as a control. Cells were cultured in normoxic or hypoxic conditions for 48 h following transfection. CM were collected and spun down at 300 g for 3 min to remove cell debris and dead cells. JAK inhibitor I (#420099; Millipore, Burlington, MA, USA), JAK2 inhibitor II (#420132; Millipore) and STAT5 inhibitor (#573108; Millipore) were reconstituted in DMSO upon delivery, aliquoted and stored at −20 °C. STAT3 inhibitor I (#573096; Millipore) was diluted in water, and STAT3 inhibitor II (#573097; Millipore) was diluted in ethanol upon delivery. Working stock concentrations were achieved by dilution in culture media. Cell lines were treated with indicated concentrations for 48 h at 37 °C, 5% CO_2_ and 21% O_2_. Recombinant IL6 (R&D system, Minneapolis, MN, USA) and IL12RB2 (R&D systems) were reconstituted in PBS upon delivery, aliquoted and stored at −20 °C. Working stock concentrations were achieved by dilution in culture media, and cell lines were treated with indicated concentrations for 5 min (IL6) or 48 h (IL12RB2) in either normoxic (21% O_2_) or hypoxic (1% CO_2_) conditions.

### 2.9 Single‐cell collection

For single‐cell collection, MCF7 cells were washed with 1× PBS, pH 7.4 (Sigma‐Aldrich), and enzymatically dissociated with 0.25% trypsin‐EDTA (PAA). Cells were resuspended in 1× PBS, pH 7.4, supplemented with 2% BSA (Sigma‐Aldrich) and kept at 4 °C until sorting. Cell aggregates were removed by filtering through a 35‐μm cell strainer (BD Biosciences). Individual cells were sorted using the Becton Dickinson FACS ARIA II into 96‐well PCR plates (Life Technologies), containing 5 μL, 1 μg·μL^−1^ BSA and 2.5% glycerol (Thermo Scientific, Waltham, MA, USA) in DNase/RNase‐free water (Life Technologies). In total, 264 cells were sorted from MCF7 cells treated with HX CM from MCF7 cells, 251 cells treated with normoxia (NX) CM from MCF7 cells, 181 cells treated with HX CM from MDA‐MB 231 cells and 272 cells treated with NX CM from MDA‐MB cells.

### 2.10 Single‐cell reverse transcription

Single‐cell cDNA synthesis was performed using TATAA GrandScript cDNA Synthesis Kit (TATAA Biocenter, Gothenburg, Sweden). Briefly, 2 μL 5× TATAA GrandScript RT Reaction Mix, 0.5 μL TATAA GrandScript RT Enzyme and 2.5 μL water were added to each sample for a final volume of 10 μL. The following thermal program was used: 22 °C for 5 min, 42 °C for 30 min and 85 °C for 5 min, performed on a T100 Thermal Cycler (Bio‐Rad, Hercules, CA, USA). Samples were diluted with 30 μL DNase/RNase‐free water (Life Technologies) and stored at −20 °C.

### 2.11 Preamplification

Targeted preamplification was performed in 50 μL reactions, using iQ Supermix (Bio‐Rad), 40 nm of each primer and 20 μL diluted cDNA. Identical primer pairs were applied for targeted preamplification and downstream quantitative PCR (qPCR; Table S1). The following thermal profile was applied on a T100 Thermal Cycler (Bio‐Rad) or a PTC‐200 (MJ Researc, Waltham, MA, USAh): 95 °C for 3 min, followed by 20 cycles of amplification (95 °C for 20 s, 60 °C for 3 min and 72 °C for 20 s). During the final extended (10 min) elongation step, the samples were immediately frozen on dry ice, slowly thawed on ice, diluted 1 : 20 in 10 mm Tris and 1 mm EDTA, pH 8.0 (Life Technologies) and stored at −20 °C until analysis.

### 2.12 Single‐cell qPCR

Single‐cell qPCR was performed in 6 μL reactions using the 2× SYBR GrandMaster Mix (TATAA Biocenter), 400 nm of each primer and 3 μL diluted preamplified cDNA as template. qPCRs were carried out in white 384‐well plates (Bio‐Rad) applying the following thermal protocol: 95 °C for 1 min: 40 cycles of: 95 °C for 5 s, 60 °C for 20 s, 70 °C for 20 s (CFX384 Touch Real‐Time PCR Detection System; Bio‐Rad). Cycles of quantification (Cqs) were determined by the second‐derivative maximum method (cfx manager software, Bio‐Rad, version 3.0). All qPCR experiments were conducted in accordance with the MIQE guidelines (Bustin *et al*., 2009). Experimental details unique for single‐cell analysis are described elsewhere (Stahlberg *et al*., 2012, 2013).

### 2.13 Ethics approval and consent to participate

Breast cancer tissue was obtained with written informed consent through Sahlgrenska University Hospital, and all patients gave written consent for the use of material for research purposes. The study was approved by the Regional Ethic Authority in Gothenburg (reference #515‐12), and methodologies conformed to the standards set by the Declaration of Helsinki. Mice were housed in Experimental Biomedicine Animal Unit, University of Gothenburg, and the study was approved by the Animal Research Ethical Committee of Gothenburg, and proper animal experimentation guidelines were followed.

### 2.14 Statistical analysis

All statistical analyses were carried out using graphpad prism 7.0 Software (GraphPad, San Diego, CA, USA). For all tests, data were considered significant if *P < 0.05, **. *P *<* *0.01 and *** *P *<* *0.001. Two‐tailed unpaired and paired Student′s *t*‐test was used to calculate differences in mammosphere formation and gene expression. For cytokine analysis, cytokines were considered to be overexpressed if NX‐HX > 1, unaltered if NX‐HX = 0 and underexpressed if HX‐NX > 1. Mammosphere formation data were presented with error bars showing standard deviation (SD) and single‐cell gene expression with error bars showing standard error of means (SEM). Pearson′s chi‐square test was used to calculate significance between percentage positive cells in three different groups; differentiation positive/pluripotency negative, double positive for differentiation and pluripotency and differentiation negative/pluripotency positive in the gene expression data from the single‐cell analysis.

## 3 Results

### 3.1 Diverse response of hypoxia‐induced secretion depends on ERα status of breast cancer cells

**Figure 1:**
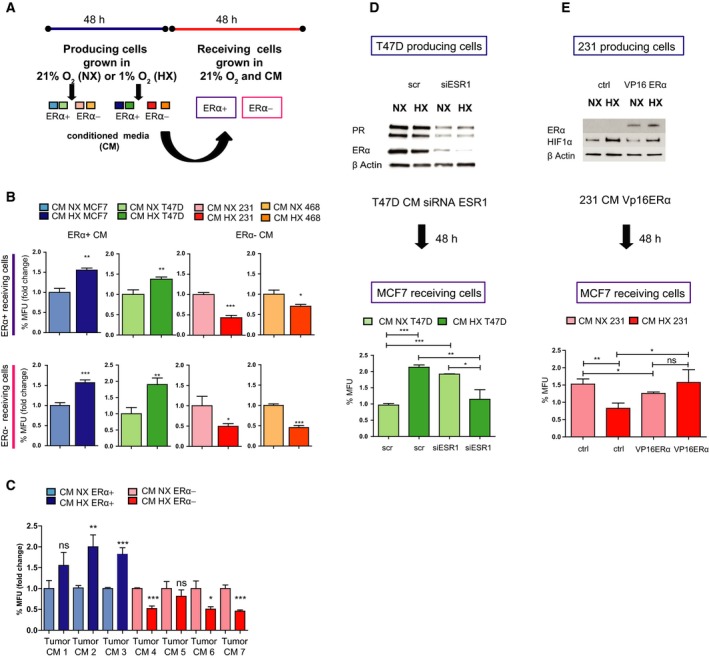
HX‐induced secretion spreads contrasting effects on mammosphere formation and tumour take dependent on ER‐α status. (A) Illustration of experimental set‐up. Detailed description in Material and methods2 section. (B) Mammosphere formation analysis was performed on ERα‐positive (MCF7 and T47D cells) and ERα‐negative cells (MDA‐MB 231 and MDA‐MB 468 cells) after 48‐h treatment with CM from ERα‐positive (MCF7) and ERα‐negative (MDA‐MB‐231) cells cultured for 48 h in NX (21% oxygen) and HX (1% oxygen). Results are expressed as relative mammosphere formation ± SD normalized to control (NX), and statistical significance was tested using unpaired *t*‐test (*n* = 3). *P < 0.05, **P <* *0.01 and ***P <* *0.001. (C) Mammosphere‐forming capacity was analysed in MCF7 cells treated for 48 h with CM from patient‐derived material after 48‐h culturing on dental sponges in NX and HX. Results are expressed as relative mammosphere formation ± SD, and statistical significance was tested using unpaired *t*‐test (*n* = 3). *P < 0.05, **P <* *0.01 and ***P <* *0.001. (D) Western blot determination of ERα after siRNA knockdown of the *ESR1* gene or scr control in T47D cells. Mammosphere formation assay was carried out on MCF7 receiving cells to verify the effect of CM from siESR1‐knockdown cells. Results are expressed as relative mammosphere formation ± SD, and statistical significance was tested using unpaired *t*‐test (*n* = 3). *P < 0.05, **P <* *0.01 and ***P <* *0.001. (E) Transient overexpression of ERα using a vp16 ERα plasmid and empty vector as control in MDA‐MB 231 cells followed by 48‐h normoxic and hypoxic incubation. Mammosphere assay in MCF7 receiving cells treated with CM from MDA‐MB 231 with overexpression of ERα. Results are expressed as relative mammosphere formation ± SD, and statistical significance was tested using unpaired *t*‐test (*n* = 3).). *P < 0.05, **P <* *0.01 and ***P <* *0.001.

To delineate the potential aspect of an extended hypoxic signalling via secretion, two ERα‐positive cell lines, MCF7 and T47D, and two ERα‐negative cell lines, MDA‐MB 231 and MDA‐MB 468, were treated with CM from cells that had been grown under hypoxic or normoxic conditions for 48 h (Fig. 1A). Interestingly, CM from hypoxic ERα‐positive cells increased the mammosphere‐forming capacity in the recipient cells regardless of their own ERα status. In contrast, cells treated with CM from ERα‐negative hypoxic cells showed a decrease in mammosphere formation (Fig. 1B). In addition, to further validate the mammosphere‐forming capacity, the morphology of cell colonies was analysed as it has previously been shown that holoclones, which are round or oval in shape with tightly packed cells, contain the CSC subpopulation (Liu *et al*., 2013). Results show that the ability to form holoclones showed similar results as the mammosphere data with a significant increased response to hypoxic CM from ERα‐positive treatment (Fig. S1A,B. Compare NX CM vs. HX CM scramble (scr) control). Next, we used patient‐derived material from ERα‐positive and ERα‐negative breast cancer patients to confirm our previous findings. Tumour biopsies were sectioned and incubated on a gelatine sponge in HX or NX for 48 h followed by collection of CM. Results showed that MCF7 cells treated with CM from patient‐derived tissue section cultures had similar effects on mammosphere formation as when using CM from breast cancer cell lines (Fig. 1C) with a pronounced contrasting response to hypoxic CM from ERα‐positive versus ERα‐negative patient‐derived tissue cultures. Data from tumour formation ability in mice, as a measure of the CSC presence, were nevertheless less conclusive. There was only a significant reduction (*P *=* *0.022) in tumour take using ERα‐negative hypoxic patient‐derived CM treatment.

Moreover, to determine whether HX‐linked secretion is directly dependent on the expression of ERα, siRNA specifically targeting *ESR1* was used. When using CM from ERα‐knockdown cells, the mammosphere‐forming capacity as well as holoclone formation in recipient cells was reversed, mimicking the ERα‐negative behaviour and suggesting a direct link between ERα‐ and HX‐dependent secretion (Figs 1D and S1). In addition, overexpressing ERα in the ERα‐negative cell line MDA‐MB 231 results in a significant change on the effect of hypoxic secretion. However, ERα overexpression did not fully revert the ERα‐negative behaviour to an ERα‐positive response (Fig. 1E).

### 3.2 Increased pluripotency signature of cells cultured in hypoxic conditioned media from ERα‐positive breast cancer cells

**Figure 2:**
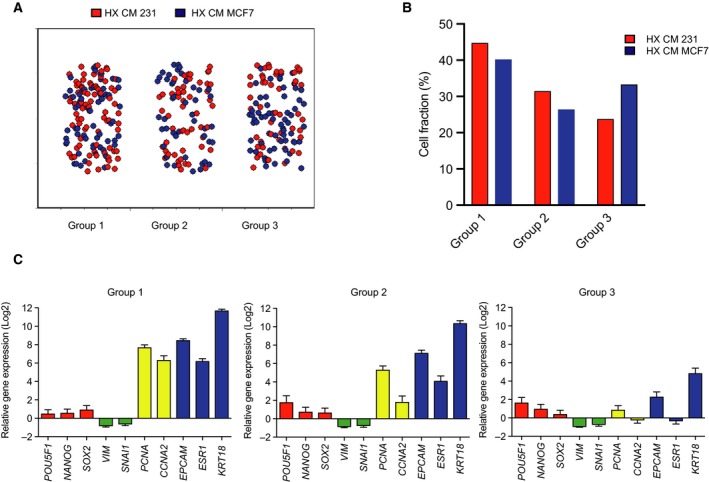
MCF7 cells exposed to hypoxic CM promote an accumulation of stem cell‐like cell behaviour on single‐cell level. (A) Self‐organizing map of MCF7 single cells treated with HX CM from ERα‐negative (MDA‐MB 231) and ERα‐positive (MCF7) cells. (B) Distribution and percentage of cells from each treatment in each SOM group. (C) Single‐cell gene expression profile in each SOM group expressed as relative gene expression with SEM.

To define and characterize subgroups of cells after treatment with CM from various cultures, we used single‐cell gene expression profiling applying qPCR. In order to delineate subsets of cellular differentiation stages, we analysed genes involved in pluripotency (*POU5F1, NANOG* and *SOX2*), epithelial‐to‐mesenchymal transition (EMT; *VIM* and *SNAI1*), proliferation (*PCNA* and *CCNA2*) and differentiation (*KRT18, ESR1* and *EPCAM*) as earlier established (Akrap *et al*., 2016). The overall effect of ERα‐positive CM treatment showed a significant increased expression of *POU5F1* in cells treated with hypoxic CM compared to normoxic CM and a significant decrease in *ESR1* expression (Fig. S2A). These observations support the hypothesis of either an expansion of the *POU5F1*‐expressing subpopulation or a loss of differentiation. To define gradual expression changes, we used a self‐organizing map algorithm (SOM). Results show that after ERα‐negative versus ERα‐positive hypoxic CM treatment, cells subdivide into three SOM groups when using the expression of genes involved in pluripotency, EMT, proliferation and differentiation (Fig. 2A). In addition, we observed that there was an overrepresentation of cells treated with ERα‐negative hypoxic CM in SOM group 1, whereas there was an overrepresentation of ERα‐positive hypoxic CM‐treated cells in SOM group 3 (Fig. 2B). The gene expression in each SOM group revealed that cells treated with ERα‐negative hypoxic CM illustrate a more differentiated and proliferating cell population while treatment with ERα‐positive hypoxic CM demonstrates decreased differentiation and an increase in pluripotency signature (Fig. 2C). Moreover, the overall effect on the ERα‐positive MCF7 cells treated with CM from the ERα‐negative MDA‐MB 231 cells showed no significant differences on individual gene level (data not shown) or difference in clustering using distribution analysis (Fig. S2B). However, when we correlate the index group differentiation and pluripotency and further separate the population into differentiation positive/pluripotency negative, differentiation negative/pluripotency positive, double positive and double negative, we observed a significant change where the hypoxic CM treatment decreased the differentiation negative/pluripotency positive group (Fig. S2C), suggesting a loss of the most primitive, stem‐like cells.

### 3.3 The JAK‐STAT pathway is involved in regulating the contrasting secretome of ERα‐positive and ERα‐negative breast cancer cells

**Figure 3:**
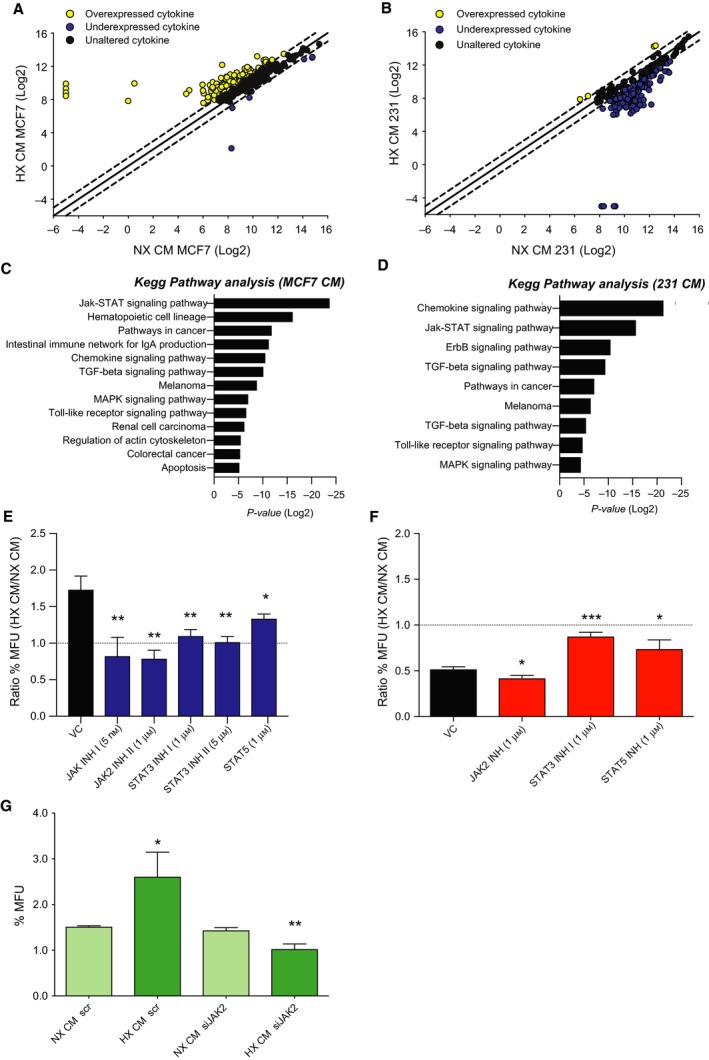
Cytokine secretion highlights the JAK‐STAT pathway as a key regulator in response to HX. (A) Correlation plot of cytokine array units (log 2) between HX and NX CM from MCF7 cells. Each point represents one cytokine value. Points outside the dotted line (95% confidence interval) were considered to be notably changed. Overexpressed (yellow, NX‐HX > 1), unaltered (black, NX‐HX = 0) and underexpressed (blue, HX‐NX > 1) cytokines. (B) Correlation plot of cytokine array units (log 2) between HX CM and NX CM from MDA‐MB 231 cells. Each point represents one cytokine log 2 values. Points outside the dotted line were considered to be notably changed. Overexpressed (yellow), unaltered (black) and underexpressed (blue) cytokines. (C, D) Biological processes involving the identified secreted proteins significantly changed between NX CM and HX CM from MCF7 (left) and MDA‐MB 231 cells (right). (E, F) Mammosphere‐forming units presented as ratio between MCF7 and MDA‐MB 231 cells treated with HX CM and NX CM from MCF7 (left) and MDA‐MB 231 cells (right) after 48 h of inhibitor treatment. Results are expressed as relative mammosphere formation ± SD, and the significant differences in ratio were calculated between vehicle control (VC) treatment and each inhibitor using unpaired *t*‐test (*n* = 3). *P < 0.05, **P <* *0.01 and ***P <* *0.001. (G) MCF7 cells treated with CM from siRNA knockdown of JAK2 or scr in T47D cells for 48 h. Results are expressed as relative mammosphere formation ± SD, and statistical significance was tested using unpaired *t*‐test (*n* = 3) *P < 0.05, **P <* *0.01 and ***P <* *0.001.

In order to define potential differences in cytokine secretion during hypoxic conditions from the various cell lines used, we screened the CM using a human biotin label‐based cytokine antibody array with 507 human proteins. From this screen, 165 cytokines were identified as upregulated (NX‐HX > 1) whilst 6 cytokines were reduced (HX‐NX > 1) in hypoxic CM versus normoxic control media from MCF7 cells. Interestingly, CM from MDA‐MB 231 cells showed an overall reduction of secreted factors after HX, with 174 reduced cytokines and only four increased cytokines (Fig. 3A, B). Further, the significantly altered cytokine hits were analysed using the KEGG pathway enrichment software to identify potentially enriched pathways involved in the regulation of CSCs. The fact that we observed that the mammosphere‐forming capacity had contrasting effects dependent on ERα status of the secreting and hypoxic stimulated cells, we hypothesized that the hypoxic secretome from ERα‐positive and ERα‐negative cells is significantly different. Interestingly, the pathway analysis identified the JAK‐STAT pathway in both the ERα‐positive and ERα‐negative hypoxic CM, suggesting that this pathway could play a pivotal role in influencing the BCSC population (Figs 3C,D and S3A,B). To validate the role of JAK‐STAT activation on the mammosphere‐forming capacity, we used a panel of inhibitors targeting both JAK and STAT. Results showed that both JAK and STAT inhibitors reduce the HX‐mediated increase in mammosphere formation by ERα‐positive MCF7 CM (Fig. 3E), whereas only the STAT inhibitors among the JAK‐STAT inhibitors tested for ERα‐negative MDA‐MB 231 CM treatment (Fig. 3F). Further, silencing JAK2 using siRNA in the recipient cells followed by CM treatment demonstrates an important role for this signalling pathway in mammosphere‐forming capacity (Fig. 3G).

### 3.4 Hypoxia‐induced IL6‐mediated STAT3 phosphorylation in breast cancer cells

**Figure 4:**
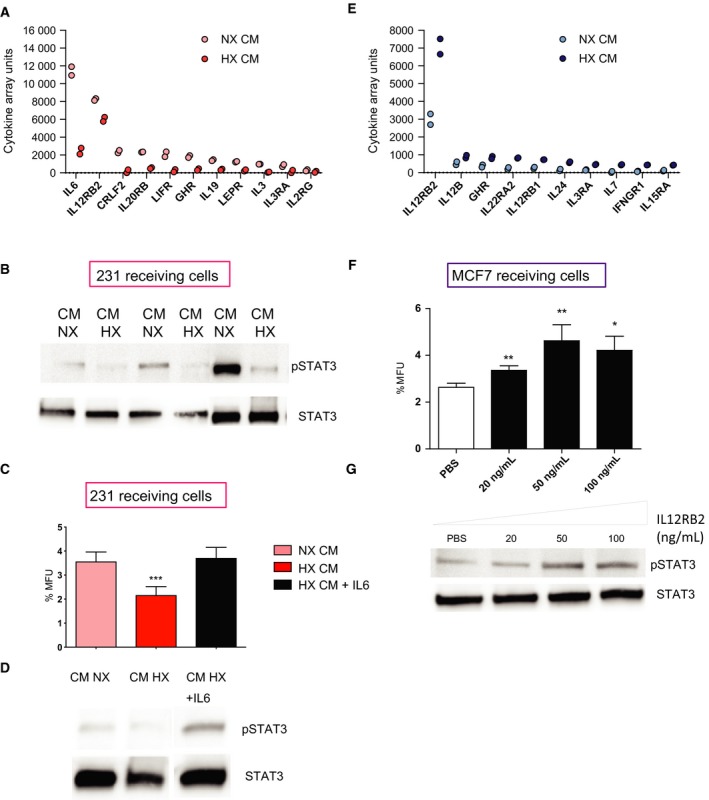
STAT3 is involved in both ERα‐positive and ERα‐negative hypoxic response in opposing directions. (A) Representation of cytokines involved in the JAK‐STAT pathway that were altered between NX and HX CM from MDA‐MB 231 cells. (B) CM from three ERα‐negative explants incubated under NX and HX conditions for 48 h were used to treat the ERα‐negative MDA‐MB 231 receiving cells and analysed for phosphorylation at tyrosine 705 STAT3. (C, D) MDA‐MB 231 cells were treated with NX, HX CM from MDA‐MB 231 cells or HX CM from MDA‐MB 231 cells with addition of human recombinant IL6 (100 ng·mL^−1^) for 5 min (for IL6 treatment) and 1 h (for CM treatment) and analysed for mammosphere‐forming capacity and by western blot for activation of STAT3. Results are expressed as relative mammosphere formation ± SD, and statistical significance was tested using unpaired *t*‐test (*n* = 3). *P < 0.05, **P <* *0.01 and ***P <* *0.001. (E) Representation of cytokines involved in the JAK‐STAT pathway that were altered between NX and HX CM from MCF7 cells. (F) MCF7 cells were treated with human recombinant IL12RB2 at various concentrations for 48 h followed by mammosphere‐forming assay for 5 days. Results are expressed as relative mammosphere formation ± SD, and statistical significance was tested using unpaired *t*‐test (*n* = 3). *P < 0.05, **P <* *0.01 and ***P <* *0.001. (G) Phosphorylated STAT3 was measured using western blot after 48 h treatment. Total STAT3 was used as a control.

Based on the cytokine array data, one of the most striking differences in cytokine secretion by the MDA‐MB 231 cells was IL6, which demonstrated a significant (*P*: 0.0104) reduction in hypoxic CM compared to normoxic CM (Fig. 4A). Further, we analysed the activation of downstream targets of IL6 and we could show that MDA‐MB 231 cells treated with hypoxic CM from ERα‐negative patient‐derived explants demonstrate less phosphorylation of STAT3 (Fig. 4B), suggesting an IL6‐mediated phosphorylation of STAT3. In addition, to verify the role of IL6 we treated MDA‐MB 231 cells with hypoxic CM from MDA‐MB 231 cells with addition of IL6. Results show that the HX‐mediated decrease in mammosphere‐forming capacity with ERα‐negative CM treatment was rescued with the addition of IL6 (Fig. 4C) along with induction of pSTAT3 (Fig. 4D).

Moreover, one of the most pronounced changes in ERα‐positive hypoxic CM among the JAK‐STAT‐related secreted factors was an increase in IL12RB2 (*P*: 0.0201; Fig. 4E). Therefore, we tested whether treatment with this single peptide would mimic the hypoxic behaviour mediated by ERα‐positive cells. In line with the hypothesis, we observed a significant increase in mammosphere formation in ERα‐positive MCF7 recipient cells following IL12RB2 administration (Fig. 4F). IL12RB2 is known to be a direct binding partner to STAT4. Therefore, we investigated whether STAT4 was activated after IL12RB2 treatment. Results show that there was no activation of STAT4 after IL12RB2 treatment (data not shown) but instead an increase in phosphorylated STAT3 (Fig. 4G), suggesting that the subunit IL12RB2 of the interleukin (IL)‐12 receptor is important for STAT3 activation.

## 4 Discussion

Microenvironmental factors such as HX strongly affect tumour cells, participating in driving tumour progression, and influence the response to treatment (Bustin *et al*., 2009; Dean *et al*., 2012). In this study, we examined whether HX influences subtypes of breast cancer. Our results show that CM from hypoxic ERα‐positive breast cancer cells increased the fraction of CSCs compared to normal growth conditions, whereas media from ERα‐negative hypoxic cells instead decreased this subpopulation of cancer cells. Further, we could show that JAK‐STAT‐associated cytokines are involved in both the ERα‐positive and ERα‐negative linked hypoxic processes as determined by a cytokine screen of the CM. JAK‐STAT inhibitors and knockdown experiments further supported that this pathway was critical for the contrasting CSC response in the ERα positive and ERα negative induced by hypoxic secretion. The JAK‐STAT pathway was originally discovered in a study of interferon signalling, identifying how a growth factor leads to the activation of a transcription factor (Kiu and Nicholson, 2012). Cytokines, receptors, tyrosine kinases and protein inhibitors among other factors tightly regulate JAK‐STAT activation. In contrast to normal cells, STAT1, 3, 4 and 5 are constitutively tyrosine phosphorylated in most malignancies, suggesting that this pathway play an important role in disease progression.

Based on the cytokine screen, the most striking differences in cytokine secretion from ERα‐negative MDA‐MB 231 cells exposed to hypoxic environment was seen in IL6, which showed a strong reduction in hypoxic CM compared to normoxic CM. This suggests that IL6 may be involved in the ERα‐negative behaviours during hypoxic conditions, where we observed a decrease in stem cell activity. Elevated serum IL6 has been demonstrated to associate with clinical outcome in several cancers, including breast cancer and prostate cancer where IL6 has been shown to have pleiotropic properties (Fisher *et al*., 2014). IL6 was demonstrated to both stimulate and reduce tumour cell proliferation and growth, inhibit apoptosis and enhance invasiveness and metastasis. A constitutive activation of IL6‐JAK2‐STAT3 axis has previously been described in breast cancer, especially pronounced in basal subtypes (Iliopoulos *et al*., 2011). Here, we observed that the IL6‐JAK2‐STAT3 axis was specifically central for the ERα‐positive hypoxic behaviour. Our results show that the ERα‐negative MDA‐MB 231 cells treated with hypoxic CM from ERα‐negative patient‐derived explants revealed a decreased phosphorylation of STAT3. The fact that JAK‐STAT is a downstream target of IL6 and that we observed a reduction of IL6 secretion in ERα‐negative hypoxic CM suggests an IL6‐mediated phosphorylation of STAT3. These results suggest that the reduction in activated STAT3 in hypoxic predisposed ERα‐negative cells contributes to the decrease in stem cell activity.

Moreover, the most pronounced change in ERα‐positive CM among the JAK‐STAT‐related secreted factors was an increase in IL12RB2. IL12RB2 is a subunit of the IL‐12 receptor, which together with IL12RB1 strongly bind IL‐12. Previously, IL12RB2 has been reported as a direct binding partner to STAT4 as well as being required for activation of the STAT4 pathway (Yao *et al*., 1999). When examining this potential association with STAT4, there was nevertheless no activation of STAT4 after IL12RB2 treatment but instead a significant increase in phosphorylated STAT3. Collectively, the IL6 and IL12RB2 treatment data suggest that STAT3 signalling plays a role in both ERα‐negative behaviour and ERα‐positive behaviour in hypoxic conditions but in opposite directions.

## 5 Conclusion

Altogether, our data support a dynamic system that is dependent on communication between the different cell types through secretion to maintain the CSC niche. We show that BCSCs from ERα‐negative and ERα‐positive breast cancer are inversely influenced by HX‐induced cytokine secretion, which highlights the importance of HX‐induced secretion in spreading contrasting BCSC influencing signalling in subtypes of the disease. However, the HIF‐α and HIF‐α paralog associations to HX‐induced CSC propagating secretion should nevertheless be explored in more detail. Our results also underline the importance of considering breast cancer subtypes in treatments targeting JAK‐STAT or HX‐associated processes and highlight that HX is not only a confined tumour biological event in cancer but will influence key tumour properties in widespread normoxic microenvironments.

## Conflict of interest

AS is a shareholder of TATAA Biocenter. No other potential conflicts of interest were disclosed.

## Author contributions

HJ, GL and HH conceptualized the study; HJ, HH, ÉH, PF, DA, AS and GL involved in methodology; HJ, HH, ÉH, EP, PF, PG, AG, YM and DA contributed to investigation; H.J contributed to writing the original draft of the manuscript; HJ, HH, EP, SR, PF, HJ, AS, DA and GL contributed to writing, that is review and editing, the original draft of the manuscript; G.L involved in funding acquisition; GL and AS provided resources; and G.L supervised the study.

## Supporting information


**Fig. S1**. (a) MCF7 and (b) T47D were transfected with siRNA against *ESR1* or scr control followed by 48‐h incubation in normoxic (NX) and hypoxic (HX) conditions. Progesterone expression levels were used as a functional control for the siESR1 knockdown. A holoclone assay was carried out in MCF7 and MDA‐MB 231 receiving cells treated with CM from siESR1 knockdown MCF7 or T47D cells. Results are expressed as relative holoclone formation ± SD and statistical significance was tested using unpaired *t*‐test (*n* = 3). *P < 0.05, **P <* *0.01 and ***P <* *0.001 (c) Image of MCF7 and MDA‐MB 231 holoclone. Scale bar represent 100 μm.

**Fig. S2**. (a) Descriptive statistics of MCF7 cells treated with normoxic (NX) and hypoxic (HX) CM from MCF7 cells for 48 h. Statistical significance was tested using unpaired *t*‐test between NX CM (bright blue) treated MCF7 cells (*n* = 251) and HX CM (dark blue) treated MCF7 cells (*n* = 264) and presented with SEM. *P < 0.05. (b) Correlation plot for MCF7 cells treated with MDA‐MB 231 CM NX (bright red) and 231 CM HX (red) between differentiation genes and pluripotency genes. (c) A comparison between NX CM and HX CM treated MCF7 cells presented as percentage positive cells in three different groups; Differentiation positive/pluripotency negative, double positive for differentiation and pluripotency and differentiation negative/pluripotency positive. Statistical significance was tested using Chi square test. **P <* *0.01.

**Fig. S3**. Biological processes involving the identified secreted proteins significantly changed between NX CM and HX CM from MDA‐MB 468 (a) and T47D cells (b).

**Table S1.** Primer pairs.

## References

[mol212500-bib-0001] Akrap N , Andersson D , Bom E , Gregersson P , Ståhlberg A and Landberg G (2016) Identification of distinct breast cancer stem cell populations based on single‐cell analyses of functionally enriched stem and progenitor pools. Stem Cell Reports 6, 121–136. 26771357 10.1016/j.stemcr.2015.12.006 PMC4719187

[mol212500-bib-0002] Bak Y , Kwon T , Bak IS , Hong J , Yu DY and Yoon DY (2016) IL‐32theta inhibits stemness and epithelial‐mesenchymal transition of cancer stem cells via the STAT3 pathway in colon cancer. Oncotarget 7, 7307–7317. 26824417 10.18632/oncotarget.7007 PMC4872787

[mol212500-bib-0003] Bustin SA , Benes V , Garson JA , Hellemans J , Huggett J , Kubista M , Mueller R , Nolan T , Pfaffl MW , Shipley GL *et al* (2009) The MIQE guidelines: minimum information for publication of quantitative real‐time PCR experiments. Clin Chem 55, 611–622. 19246619 10.1373/clinchem.2008.112797

[mol212500-bib-0004] Campbell LL and Polyak K (2007) Breast tumor heterogeneity: cancer stem cells or clonal evolution? Cell Cycle 6, 2332–2338. 17786053 10.4161/cc.6.19.4914

[mol212500-bib-0005] Centenera MM , Raj GV , Knudsen KE , Tilley WD and Butler LM (2013) *Ex vivo* culture of human prostate tissue and drug development. Nat Rev Urol 10, 483. 23752995 10.1038/nrurol.2013.126

[mol212500-bib-0006] Criscitiello C , Esposito A and Curigliano G (2014) Tumor‐stroma crosstalk: targeting stroma in breast cancer. Curr Opin Oncol 26, 551–555. 25279962 10.1097/CCO.0000000000000122

[mol212500-bib-0007] Dean JL , McClendon AK , Hickey TE , Butler LM , Tilley WD , Witkiewicz AK and Knudsen ES (2012) Therapeutic response to CDK4/6 inhibition in breast cancer defined by *ex vivo* analyses of human tumors. Cell Cycle 11, 2756–2761. 22767154 10.4161/cc.21195 PMC3409015

[mol212500-bib-0008] Di Stefano AB , Iovino F , Lombardo Y , Eterno V , Hoger T , Dieli F , Stassi G and Todaro M (2010) Survivin is regulated by interleukin‐4 in colon cancer stem cells. J Cell Physiol 225, 555–561. 20506498 10.1002/jcp.22238

[mol212500-bib-0009] Fisher DT , Appenheimer MM and Evans SS (2014) The two faces of IL‐6 in the tumor microenvironment. Semin Immunol 26, 38–47. 24602448 10.1016/j.smim.2014.01.008 PMC3970580

[mol212500-bib-0010] Fuchs E , Tumbar T and Guasch G (2004) Socializing with the neighbors: stem cells and their niche. Cell 116, 769–778. 15035980 10.1016/s0092-8674(04)00255-7

[mol212500-bib-0011] Gilkes DM , Semenza GL and Wirtz D (2014) Hypoxia and the extracellular matrix: drivers of tumour metastasis. Nat Rev Cancer 14, 430–439. 24827502 10.1038/nrc3726 PMC4283800

[mol212500-bib-0012] Ginestier C , Charafe‐Jauffret E and Birnbaum D (2011) Breast tumor microenvironment: in the eye of the cytokine storm. Cell Cycle 10, 2420–2421. 21734456

[mol212500-bib-0013] Ginestier C , Liu S , Diebel ME , Korkaya H , Luo M , Brown M , Wicinski J , Cabaud O , Charafe‐Jauffret E , Birnbaum D *et al* (2010) CXCR14 blockade selectively targets human breast cancer stem cells *in vitro* and in xenografts. J Clin Investig 120, 485–497. 20051626 10.1172/JCI39397 PMC2810075

[mol212500-bib-0014] Harrison H , Pegg HJ , Thompson J , Bates C and Shore P (2018) HIF1‐alpha expressing cells induce a hypoxic‐like response in neighbouring cancer cells. BMC Cancer 18, 674. 29925335 10.1186/s12885-018-4577-1 PMC6011406

[mol212500-bib-0015] Harrison H , Rogerson L , Gregson HJ , Brennan KR , Clarke RB and Landberg G (2013) Contrasting hypoxic effects on breast cancer stem cell hierarchy is dependent on ER‐alpha status. Cancer Res 73, 1420–1433. 23248117 10.1158/0008-5472.CAN-12-2505

[mol212500-bib-0016] Hu M , Peluffo G , Chen H , Gelman R , Schnitt S and Polyak K (2009) Role of COX‐2 in epithelial‐stromal cell interactions and progression of ductal carcinoma *in situ* of the breast. Proc Natl Acad Sci USA 106, 3372–3377. 19218449 10.1073/pnas.0813306106 PMC2642666

[mol212500-bib-0017] Iliopoulos D , Hirsch HA , Wang G and Struhl K (2011) Inducible formation of breast cancer stem cells and their dynamic equilibrium with non‐stem cancer cells via IL6 secretion. Proc Natl Acad Sci USA 108, 1397–1402. 21220315 10.1073/pnas.1018898108 PMC3029760

[mol212500-bib-0018] Kiu H and Nicholson SE (2012) Biology and significance of the JAK/STAT signalling pathways. Growth Factors 30, 88–106. 22339650 10.3109/08977194.2012.660936 PMC3762697

[mol212500-bib-0019] Korkaya H , Liu S and Wicha MS (2011) Breast cancer stem cells, cytokine networks, and the tumor microenvironment. J Clin Invest 121, 3804–3809. 21965337 10.1172/JCI57099 PMC3223613

[mol212500-bib-0020] Li HJ , Reinhardt F , Herschman HR and Weinberg RA (2012) Cancer‐stimulated mesenchymal stem cells create a carcinoma stem cell niche via prostaglandin E2 signaling. Cancer Discov 2, 840–855. 22763855 10.1158/2159-8290.CD-12-0101 PMC3833451

[mol212500-bib-0021] Liu S , Ginestier C , Ou SJ , Clouthier SG , Patel SH , Monville F , Korkaya H , Heath A , Dutcher J , Kleer CG *et al* (2011) Breast cancer stem cells are regulated by mesenchymal stem cells through cytokine networks. Can Res 71, 614–624. 10.1158/0008-5472.CAN-10-0538 PMC3100554 21224357

[mol212500-bib-0022] Liu TJ , Sun BC , Zhao XL , Zhao XM , Sun T , Gu Q , Yao Z , Dong XY , Zhao N and Liu N (2013) CD133+ cells with cancer stem cell characteristics associates with vasculogenic mimicry in triple‐negative breast cancer. Oncogene 32, 544–553. 22469978 10.1038/onc.2012.85

[mol212500-bib-0023] Lu H , Clauser KR , Tam WL , Frose J , Ye X , Eaton EN , Reinhardt F , Donnenberg VS , Bhargava R , Carr SA *et al* (2014) A breast cancer stem cell niche supported by juxtacrine signalling from monocytes and macrophages. Nat Cell Biol 16, 1105–1117. 25266422 10.1038/ncb3041 PMC4296514

[mol212500-bib-0024] Marusyk A and Polyak K (2010) Tumor heterogeneity: causes and consequences. Biochim Biophys Acta 1805, 105–117. 19931353 10.1016/j.bbcan.2009.11.002 PMC2814927

[mol212500-bib-0025] Sansone P , Storci G , Tavolari S , Guarnieri T , Giovannini C , Taffurelli M , Ceccarelli C , Santini D , Paterini P , Marcu KB *et al* (2007) IL‐6 triggers malignant features in mammospheres from human ductal breast carcinoma and normal mammary gland. J Clin Invest 117, 3988–4002. 18060036 10.1172/JCI32533 PMC2096439

[mol212500-bib-0026] Shaw FL , Harrison H , Spence K , Ablett MP , Simoes BM , Farnie G and Clarke RB (2012) A detailed mammosphere assay protocol for the quantification of breast stem cell activity. J Mammary Gland Biol Neoplasia 17, 111–117. 22665270 10.1007/s10911-012-9255-3

[mol212500-bib-0027] Singh JK , Farnie G , Bundred NJ , Simoes BM , Shergill A , Landberg G , Howell SJ and Clarke RB (2013) Targeting CXCR28/2 significantly reduces breast cancer stem cell activity and increases the efficacy of inhibiting HER2 via HER2‐dependent and ‐independent mechanisms. Clin Cancer Res 19, 643–656. 23149820 10.1158/1078-0432.CCR-12-1063 PMC4868141

[mol212500-bib-0028] Stahlberg A , Rusnakova V , Forootan A , Anderova M and Kubista M (2013) RT‐qPCR work‐flow for single‐cell data analysis. Methods 59, 80–88. 23021995 10.1016/j.ymeth.2012.09.007

[mol212500-bib-0029] Stahlberg A , Thomsen C , Ruff D and Aman P (2012) Quantitative PCR analysis of DNA, RNAs, and proteins in the same single cell. Clin Chem 58, 1682–1691. 23014600 10.1373/clinchem.2012.191445

[mol212500-bib-0030] Tyan SW , Kuo WH , Huang CK , Pan CC , Shew JY , Chang KJ , Lee EY and Lee WH (2011) Breast cancer cells induce cancer‐associated fibroblasts to secrete hepatocyte growth factor to enhance breast tumorigenesis. PLoS ONE 6, e15313. 21249190 10.1371/journal.pone.0015313 PMC3020942

[mol212500-bib-0031] Vogelstein B , Papadopoulos N , Velculescu VE , Zhou S , Diaz LA Jr and Kinzler KW (2013) Cancer genome landscapes. Science 339, 1546–1558. 23539594 10.1126/science.1235122 PMC3749880

[mol212500-bib-0032] Yao BB , Niu P , Surowy CS and Faltynek CR (1999) Direct interaction of STAT4 with the IL‐12 receptor. Arch Biochem Biophys 368, 147–155. 10415122 10.1006/abbi.1999.1302

