# Supplementary material for: Hypoxia‐induced secretion stimulates breast cancer stem cell regulatory signalling pathways
Source: Mol Oncol. 2019 Jun 26;13(8):1693–705. doi: 10.1002/1878-0261.12500 (PMC6670019; doi:10.1002/1878-0261.12500)
Supplement: Supplementary file 1 — Fig. S1. (a) MCF7 and (b) T47D were transfected with siRNA against ESR1 or scr control followed by 48‐h incubation in normoxic (NX) and hypoxic (HX) conditions. Progesterone expression levels were used as a functional control for the siESR1 knockdown. A holoclone assay was carried out in MCF7 and MDA‐MB 231 receiving cells treated with CM from siESR1 knockdown MCF7 or T47D cells. Results are expressed as relative holoclone formation ± SD and statistical significance was tested using unpaired t‐test (n = 3). *P < 0.05, **P < 0.01 and ***P < 0.001 (c) Image of MCF7 and MDA‐MB 231 holoclone. Scale bar represent 100 μm. [file MOL2-13-1693-s001.pdf]

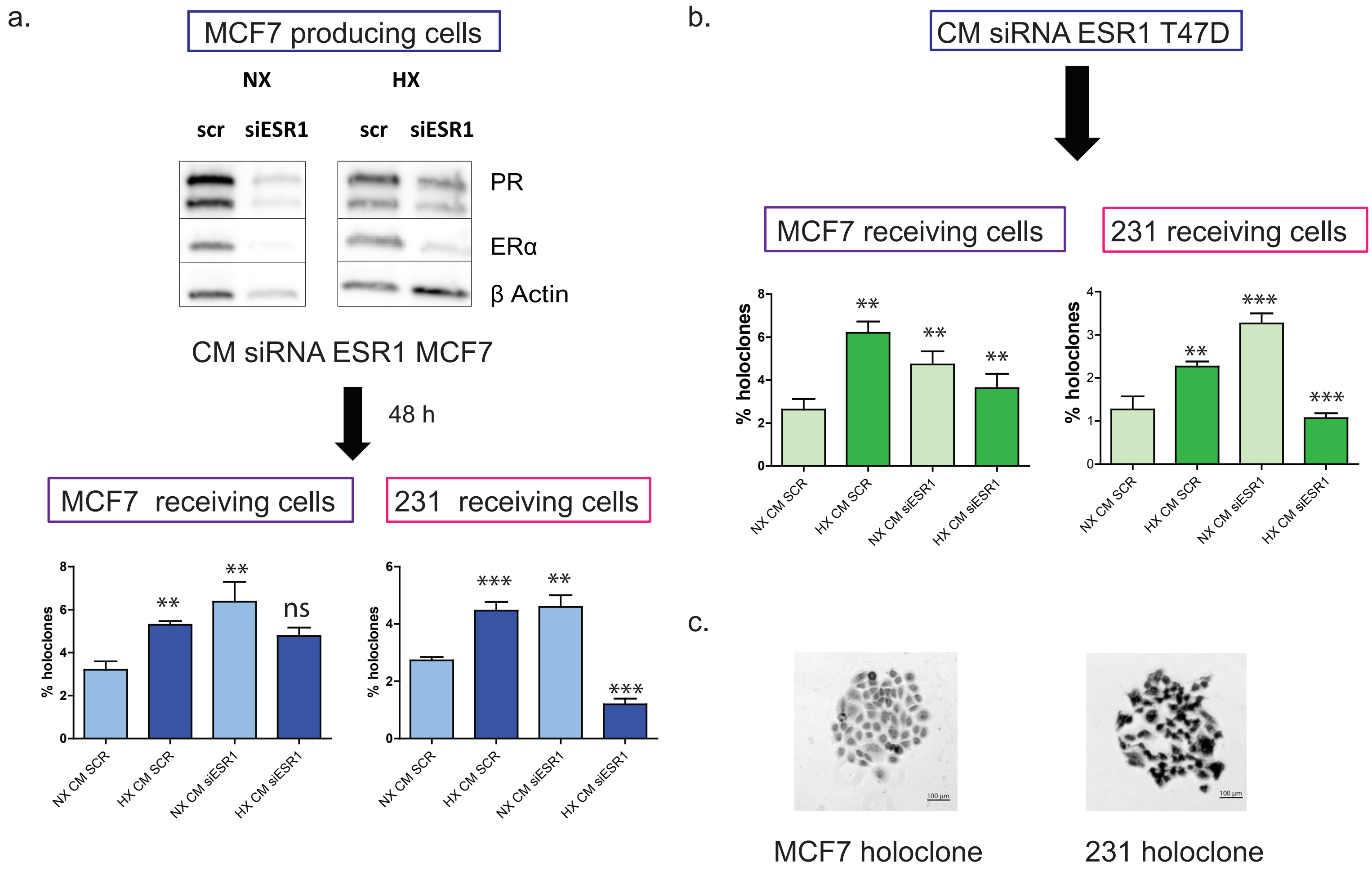
a.
MCF7 producing cells
NX
HX
scr
siESR1
scr
siESR1
PR
ERα
β Actin
CM siRNA ESR1 MCF7
48 h
MCF7 receiving cells
231 receiving cells
% holoclones
NX CM SCR
HX CM SCR
NX CM siESR1
HX CM siESR1
**
**
ns
***
**
***
b.
CM siRNA ESR1 T47D
MCF7 receiving cells
231 receiving cells
% holoclones
NX CM SCR
HX CM SCR
NX CM siESR1
HX CM siESR1
**
**
**
**
***
***
c.
MCF7 holoclone
231 holoclone
