# Supplementary figures and images for: Hypoxia‐induced secretion stimulates breast cancer stem cell regulatory signalling pathways
Source: Mol Oncol. 2019 Jun 26;13(8):1693–705. doi: 10.1002/1878-0261.12500 (PMC6670019; doi:10.1002/1878-0261.12500)

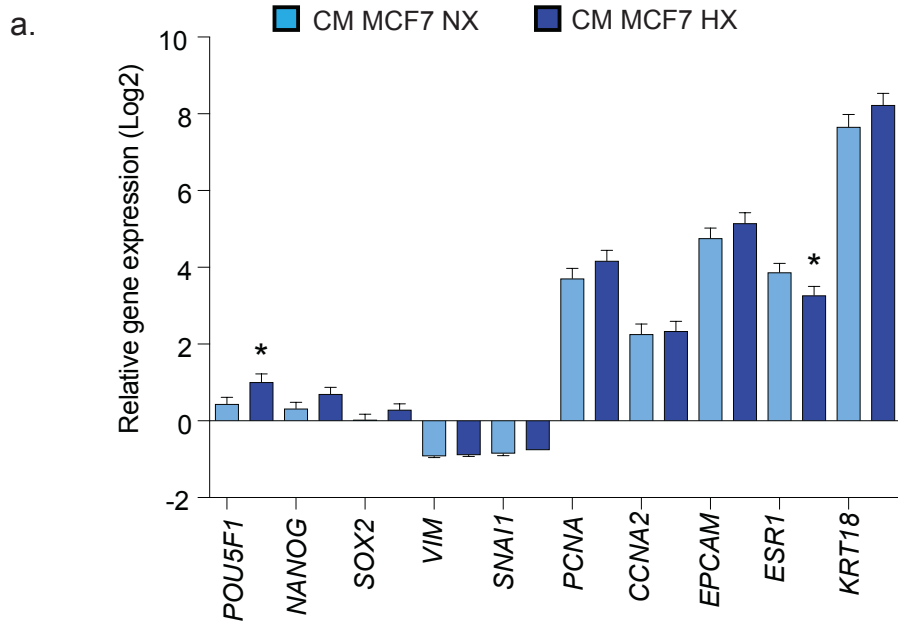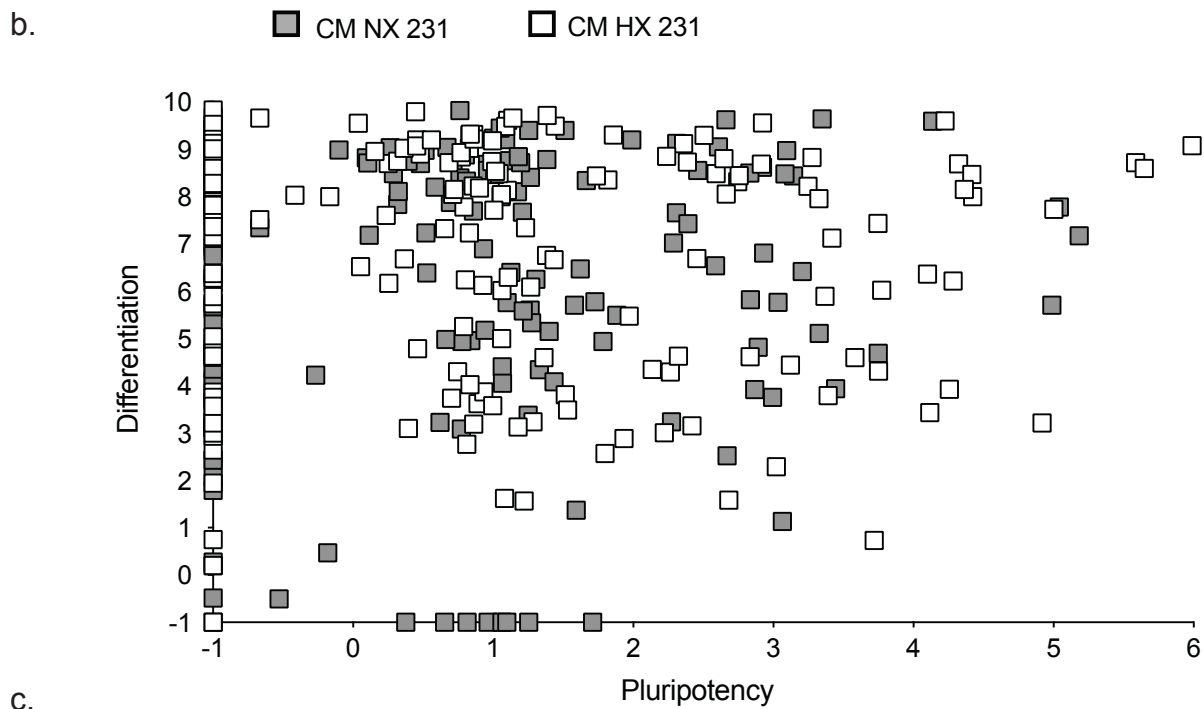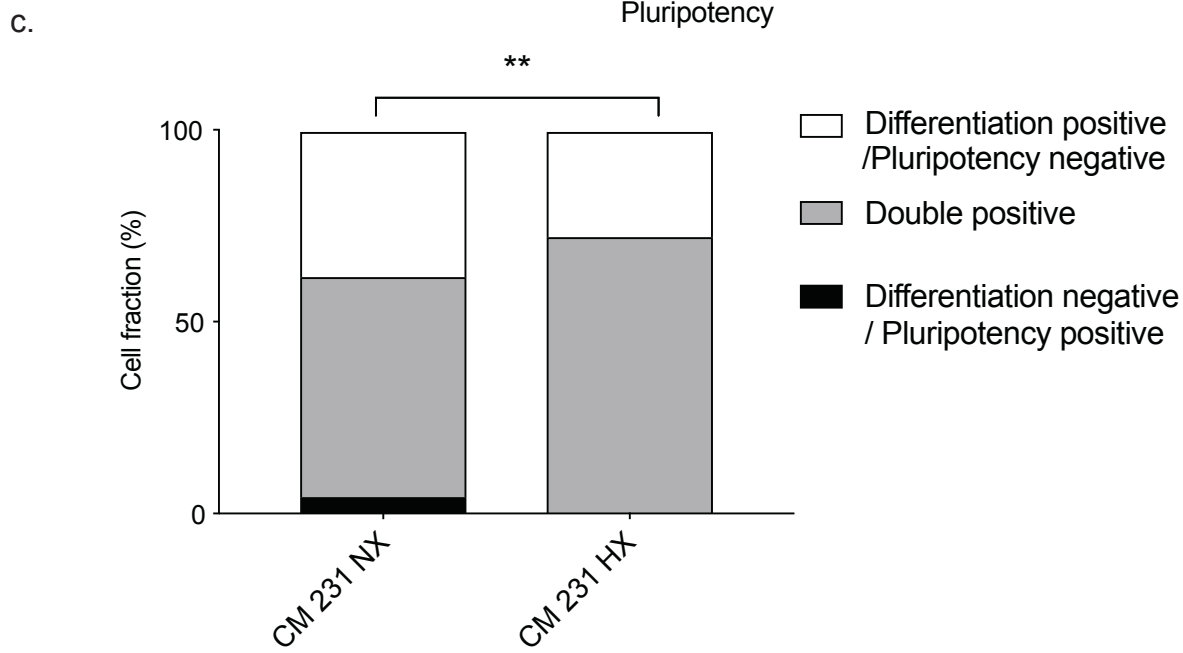

Supplement: Supplementary file 2 — Fig. S2. (a) Descriptive statistics of MCF7 cells treated with normoxic (NX) and hypoxic (HX) CM from MCF7 cells for 48 h. Statistical significance was tested using unpaired t‐test between NX CM (bright blue) treated MCF7 cells (n = 251) and HX CM (dark blue) treated MCF7 cells (n = 264) and presented with SEM. *P < 0.05. (b) Correlation plot for MCF7 cells treated with MDA‐MB 231 CM NX (bright red) and 231 CM HX (red) between differentiation genes and pluripotency genes. (c) A comparison between NX CM and HX CM treated MCF7 cells presented as percentage positive cells in three different groups; Differentiation positive/pluripotency negative, double positive for differentiation and pluripotency and differentiation negative/pluripotency positive. Statistical significance was tested using Chi square test. **P < 0.01. [file MOL2-13-1693-s002.pdf]

a.

### Kegg Pathway Analysis (468 CM)

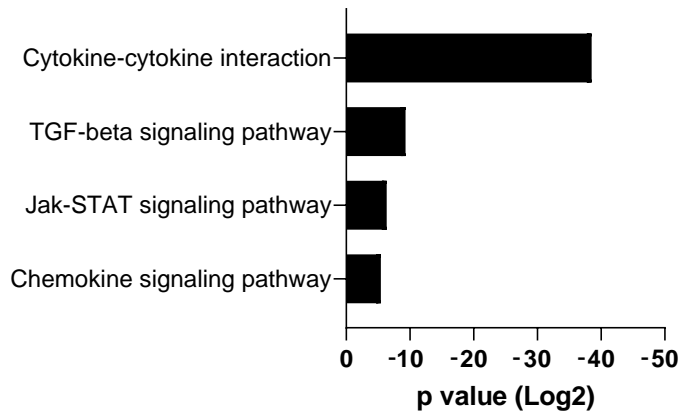

b.

### Kegg Pathway Analysis (T47D CM)

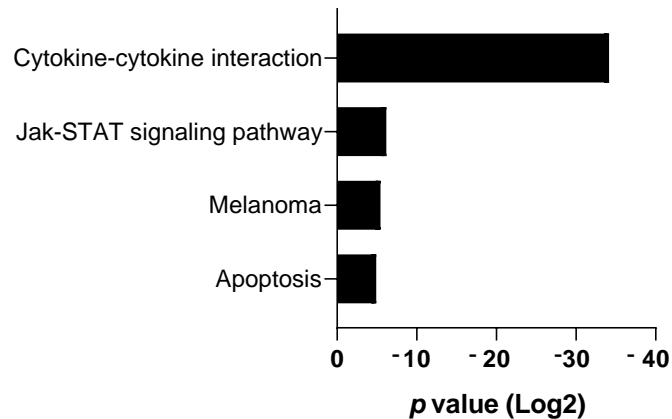

Supplement: Supplementary file 3 — Fig. S3. Biological processes involving the identified secreted proteins significantly changed between NX CM and HX CM from MDA‐MB 468 (a) and T47D cells (b). [file MOL2-13-1693-s003.pdf]
