# Supplementary material for: Hypoxia‐induced secretion stimulates breast cancer stem cell regulatory signalling pathways
Source: Mol Oncol. 2019 Jun 26;13(8):1693–705. doi: 10.1002/1878-0261.12500 (PMC6670019; doi:10.1002/1878-0261.12500)
Supplement: Supplementary file 5 [file MOL2-13-1693-s005.docx]

**Supplemental figure legends**

**Figure S1.** (**a**) MCF7 and (**b**) T47D were transfected with siRNA against *ESR1* or scrambled (scr) control followed by 48h incubation in normoxic (NX) and hypoxic (HX) conditions. Progesterone expression levels were used as a functional control for the siESR1 knockdown. A holoclone assay was carried out in MCF7 and MDA-MB 231 receiving cells treated with CM from siESR1 knockdown MCF7 or T47D cells. Results are expressed as relative holoclone formation and statistical significance was tested using unpaired t test (n=3). (**c**) Image of MCF7 and MDA-MB 231 holoclone.

**Figure S2** (**a**) Descriptive statistics of MCF7 cells treated with normoxic (NX) and hypoxic (HX) conditioned media (CM) from MCF7 cells for 48h. Statistical significance was tested using unpaired t test between NX CM (bright blue) treated MCF7 cells (n=251) and HX CM (dark blue) treated MCF7 cells (n= 264) and presented with standard error of mean (SEM). (**b**) Correlation plot for MCF7 cells treated with MDA-MB 231 CM NX (bright red) and 231 CM HX (red) between differentiation genes and pluripotency genes. (**c**) A comparison between NX CM and HX CM treated MCF7 cells presented as percentage positive cells in three different groups; Differentiation positive/pluripotency negative, double positive for differentiation and pluripotency and differentiation negative/ pluripotency positive. Statistical significance was tested using Chi square test.

**Figure S3** Biological processes involving the identified secreted proteins significantly changed between NX CM and HX CM from MDA-MB 468 (**a**) and T47D cells (**b**).
